# Assessment of prognostic role of a novel 7-lncRNA signature in HCC patients

**DOI:** 10.1016/j.heliyon.2023.e18493

**Published:** 2023-07-20

**Authors:** Junchi Qu, Di Tao, Wei Huang, Liting Lu, Junming Fan, Shineng Zhang, Fengting Huang

**Affiliations:** a Department of Gastroenterology, Sun Yat-sen Memorial Hospital, Sun Yat-sen University, Guangzhou 510120, China; b Guangdong Provincial Key Laboratory of Malignant Tumor Epigenetics and Gene Regulation, Sun Yat-Sen Memorial Hospital, Sun Yat-Sen University, Guangzhou 510120, China; c Department of Gastroenterology, The First People's Hospital of PingJiang, Yueyang 410400, China

**Keywords:** Hepatocellular carcinoma, lncRNA, Risk model, Nomogram, Immune microenvironment

## Abstract

**Background:**

Hepatocellular carcinoma (HCC) is characterized by extensive risk factors, high morbidity and mortality. Clinical prognostic evaluation assay assumes a nonspecific quality. Better HCC prognostics are urgently needed. Long noncoding RNAs (lncRNAs) exerts a crucial role in tumorigenesis and development. Excavating specific lncRNAs signature to ameliorate the high-risk survival prediction in HCC patients is worthwhile.

**Methods:**

Differentially expressed lncRNAs (DElncRNAs) profile was acquired from The Cancer Genome Atlas database (TCGA). Then, the lncRNAs high-risk survival prognostic model was established using the least absolute shrinkage and selection operator (LASSO)-Cox regression algorithm. The lncRNAs were evaluated in clinical specimen by PCR. The receiver operating characteristic curve (ROC) analysis was further conducted to assess the potential prognostic value of the model. Moreover, a visible nomogram containing clinicopathological features and prognostic model was developed for prediction of survival property. Potential molecular mechanism was assessed by GO, KEGG, GSEA enrichment analysis and CIBERSORT immune infiltration analysis.

**Results:**

A novel 7-lncRNA risk model (AL161937.2, LINC01063, AC145207.5, POLH-AS1, LNCSRLR, MKLN1-AS, AC105345.1) was constructed and validated for HCC prognosis prediction. Kaplan-Meier analysis revealed that patients in the high-risk group suffered a poor prognosis (*p* = 1.813 × 10^−8^). These genes were detected by PCR, and the expression trend was in accordance with TCGA database. Interestingly, the risk score served as an independent risk factor for HCC patients (HR: 1.166, 95% CI:1.119–1.214, *p* < 0.001). The nomogram was established, and the predictive accuracy in the nomogram was prior to the TNM stage according to the ROC curve analysis. Cell proliferation related pathway, decreased CD4^+^ T cell, CD8^+^ T cell, NK cell and elevated Neutrophil, Macrophage M0 were observed in high-risk group. Besides, suppression of MKLN1-AS expression inhibited cell proliferation of HCC cells by CCK8 assay *in vitro.*

**Conclusion:**

The 7-lncRNA signature may exert a particular prognostic prediction role in HCC and provide new insight in HCC carcinogenesis.

## 1 Introduction

Hepatocellular carcinoma (HCC) exerts the main pathological pattern of primary liver cancer which is the second cancer-related death worldwide and ranks fourth in incidence and third in mortality [[1], [2], [3]]. The prognosis of advanced HCC is devastating, with the 5-year survival rate of resection and transplantation just about 30% and 60%, respectively [2,4]. Chronic hepatitis B or C virus infection (HBV/HCV), type 2 diabetes and smoking are the high-risk factors for HCC in China [3,5,6]. It is urgently needed for an accurate clinical prognostic assessment to make more precise treatment strategy and disease course management in HCC.

Currently, the clinical prognostic evaluation assay for HCC patients mainly relies on the clinicopathological staging system. Multiple staging methods are applied in worldwide, such as Barcelona staging (BCLC), AJCC TNM staging. However, each system has its own bias and not to exist global unity, which gives rise to their certain scarcity in the prediction of survival-related prognosis during different stages of HCC progression [7,8].

To date, emerging evidences have suggested lncRNA promoted tumorigenesis and progression and predicted the outcome of cancer patients [9,10], including HCC [11]. Markedly, when compared with mRNA, lncRNAs exhibit a higher degree of time and tissue specificity [[12], [13], [14], [15]]. Moreover, it can be stably detected in patients’ plasma, cancer tissues, and body fluids [16,17], which revealed that lncRNAs can act as an independent factor to forecast the prognosis of patients HCC.

In this study, to investigate the more precise prognostic evaluation assay in HCC, a 7-lncRNA risk score model (AL161937.2, LINC01063, AC145207.5, POLH-AS1, LNCSRLR, MKLN1-AS, AC105345.1) was constructed based on the differentially expressed lncRNAs in TCGA-LIHC cohort and was validated. Then the nomogram comprising the 7-lncRNAs risk factor was established. The predictive accuracy in the nomogram showed advantage when comparing to the TNM stage. Cell proliferation related pathway, decreased CD4^+^ T cell, CD8^+^ T cell, NK cell and elevated Neutrophil, Macrophage M0 were observed in high-risk group. Besides, suppression of MKLN1-AS expression inhibited cell proliferation of HCC cells *in vitro.* It might provide an effective prognostic prediction assay and new insight on HCC treatment strategy.

## 2 Materials and methods

### 2.1 Data acquisition and preliminary processing

The transcriptome and clinical characteristics of 338 HCC cases and 50 specimens of normal tissues adjacent to cancer were downloaded from TCGA-LIHC cohort (https://portal.gdc.cancer.gov/). The data with the complete gene expression and clinical information were included. Practical Extraction and Report Language (Perl, Version 5.30.2.1) was used to help the gene name conversion and the separation of lncRNA and mRNA.

### 2.2 LncRNA model construction and validation

Differential expression of lncRNA and mRNA profiles in TCGA-LIHC cohort between cancer and normal tissues was conducted by employing the “limma” R package [18]. ｜logFc｜>1 and *p* < 0.05 were set as the differentially-expressed screening criteria. By calling the “createDataPartition” function in “caret” R package, the cancer tissue samples of 338 cases of hepatocellular carcinoma were randomly divided into training group and verification group according to the proportion of 7:3. Therefore, 238 cases in the training group were used for training to build a model, and 100 cases in verification group were used to verify the accuracy and applicability of the training model. The random grouping of this function ensures that the distribution proportion of all kinds of labels in the training group and the verification group is strictly consistent with that of the sample population (Supplementary Table 1).

By calling the “glmnet” R package, the difference lncRNA significantly related to the prognosis of hepatocellular carcinoma was screened by univariate Cox regression analysis in the training group. Then the multi-factor Cox regression model was constructed using stepwise regression, backward method [19], and the fitting degree and accuracy of each model in stepwise regression were evaluated by Akaike information criterion (AIC) [20,21] and likelihood ratios (LR) respectively. After repeated fitting, from the model with the lowest AIC value and the highest LR value, the prognosis model, namely the risk score model, was obtained. The risk score was generated as follows: Risk Score = $\sum_{1=7}^{i}Xi⨯Yi$ (X: coefficients, Y: expression value of the gene).

According to the median of risk score, the samples of training group, verification group and complete data group were divided into high risk group and low risk group, and then the time dependent receiver operating characteristic curve (timeROC) and survival curve of the subjects in these three groups were analyzed, in which the survival curve can directly reflect the difference of survival rate between different groups [22,23]. The timeROC calculated the area under the ROC curve (AUC). The commonly used time nodes were: 1 year, 3 years and 5 years. It is generally believed that AUC is low accuracy between 0.6 and 0.7, moderate accuracy between 0.7 and 0.9 and high accuracy between 0.9 and 1.0, but there may be overfitting [24]. Both of them were used to verify the prediction accuracy and applicability of the model.

### 2.3 Clinical specimens and ethical approval

The protocols implemented in the study were endorsed by the Ethics Committee of Sun Yat-sen Memorial Hospital, Sun Yat-sen University (Guangzhou, China) (SYSEC-KY-KS-2021-058). Ten pair of HCC tumor tissues and its adjacent non-tumor specimens were gathered from patients experiencing surgery in Sun Yat-sen Memorial Hospital. The samples were collected from March 2021 to April 2021 with histological confirmation. The patients received none of preoperative chemotherapy, radiotherapy or immunotherapy. All the patients signed written informed consent.

### 2.4 Quantitative real-time PCR

The expression level of the lncRNAs in the risk model was investigated in clinical specimens using qPCR. The tissue was removed from liquid nitrogen and cut into suitable size. Total RNA was disassociated and extracted with Trizol (Life Technologies, Carlsbad, CA, USA) in accordance to the manufacturer's protocol. Then the concentration and purity of RNA were determined by Nanodroop and stored at −70 °C for further study. Then synthesize cDNA according to the instructions of reverse transcriptase kit. GAPDH performed as an endogenous control. The sequence of primers was listed (Supplementary Table 2).

### 2.5 Cell culture and transfection

Human HCC cell line HepG2 was obtained from the Cell Bank of Chinese Academy of Sciences and cultured for further study. It was grown in DMEM (Boster, USA) with a concentration of 10% fetal bovine serum (FBS, Gibco, USA) in a comfortable atmosphere (at 37 °C and under 5% CO_2_).

The siRNAs against the MKLN1-AS were constructed by Genecefe Biotechnology Co., Ltd (Jiangsu, China). The siRNAs sequences were listed as follows: si-RNA-1: 5′-CAA AGA GUA UGU CGC UUA UUG UCU ATT-3′, si-RNA-2: 5′- CAG CUG GUG GUG UUU CUC UCU GAA ATT-3’.

### 2.6 Cell proliferation assay

Cell growth ability was evaluated using Cell Counting Kit-8 (CCK-8) (APExBIO, USA) assay. Cells (A total of 2 × 10^3^ per well) were seeded and grown into 96-well plates (100 μl medium per well). Then medium and CCK-8 was blended according to manufacturer and added to each well at different time points (12, 24, 36 and 48 h) after seeding. The relative absorbance was then estimated at 450 nm with use of enzyme immunoassay analyzer (Thermo MK3, USA).

### 2.7 Establishment and assessment of a nomogram prognosis prediction model

Firstly, the main clinical data of the samples were collected, including age, sex, pathological grade and AJCC TNM stage. Then the independent clinical risk factors were screened by Cox regression analysis and visualized by forest map. Then the independent clinical factors and the lncRNA risk model were combined to establish a nomogram which can directly reflect the prognosis of patients with liver cancer [25]. The accuracy and prognostic ability were evaluated by calibration curve, C-index, decision curve analyses (DCA) [26] and time-dependent ROC curve. The formula for calculating the net benefit (NB) in the DCA was as follows: $NB=\frac{tp-fp}{n}\times \frac{pt}{1-pt}$, *tp* presented the true positive number, while *fp* served as the false positive number, *n* stood for the total number of patients, *pt* meant the threshold probability.

### 2.8 Prognostic ability comparison of different prediction methods

The 1, 3 and 5-year for time-dependent ROC curves of AJCC TNM stages were drawn. The AUC values of the corresponding years were carried out on the prediction accuracy and value of the three prediction methods: nomogram, lncRNA risk model and AJCC TNM stages.

### 2.9 Enrichment analysis

The Pearson correlation analysis was conducted between the differential mRNA and the model lncRNA, and the significant correlation criterion was set as｜cor｜> 0.45，p < 0.001, so as to screen out the differential mRNA co-expressed with the model lncRNA. KEGG [27] and GO [28] were performed by using the “clusterProfler” R package [29], and the bar chart, dot chart and pathview were drawn respectively. And then Gene Set Enrichment Analysis (GSEA) [30,31] was completed. The data sets used were classical pathway (hallmarks) and cancer gene pathway.

### 2.10 Appraisal of immune infiltration and the correlation with the model

To calculate the proportions of infiltrating non-tumor cells in the tumor immune microenvironment in HCC patients, Cell-type Identification By Estimating Relative Subsets Of RNA Transcripts (CIBERSORT) [32] was utilized. Box map and stacked bar thermal map were drawn to reveal the distribution of the 22 kinds of immune cells visually. The relationship between the risk groups and the immune cells was investigated.

### 2.11 Statistical analysis

In this study, Bootstrap sampling method (createDataPartition function) for random grouping, Kaplan-Meier algorithm and logarithmic rank sum test was performed for survival evaluation. The differential expression level of lncRNAs between cancer samples and adjacent samples was analyzed by paired *t*-test. The comparison of different prediction methods was realized by normal distribution test and independent sample *t*-test. The relationship between mRNA and model lncRNA was estimated by Pearson correlation analysis, also in the correlation between the 22 kinds of immune infiltrating cells and the risk model. Paired *t*-test was also carried out to analyze the overall difference of the 22 kinds of immune infiltrating cells between high-risk and low-risk alignment. A *p* less than 0.05 was considered as significance.

## 3 Results

### 3.1 Construction of the prognostic 7-lncRNA risk model

**Fig. 1:**
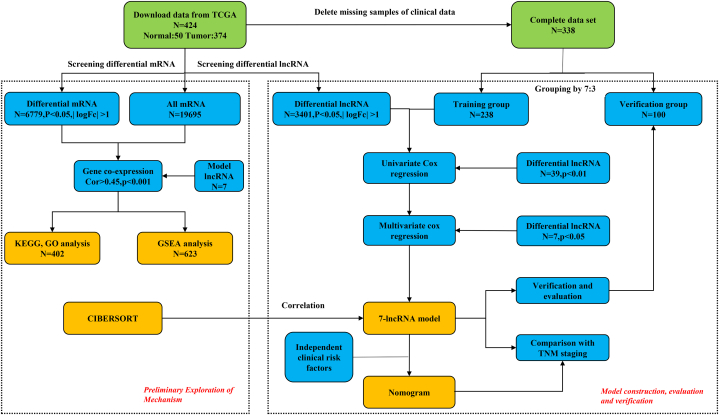
The overview of this study.

**Fig. 2:**
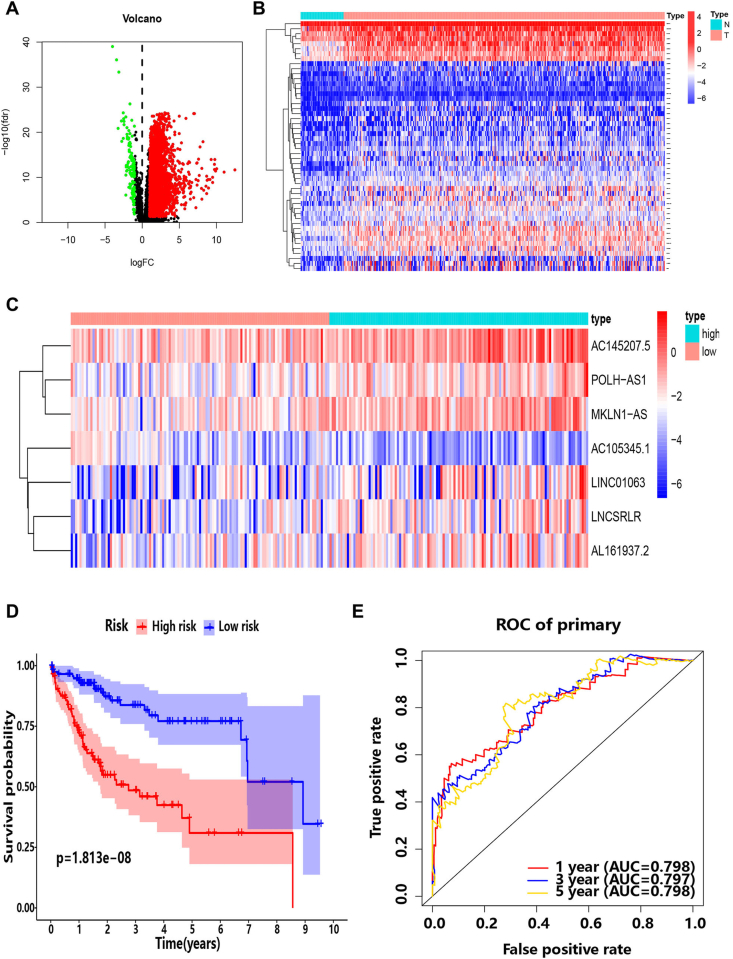
Identification of lncRNAs with significant prognostic value in HCC. (**A)** Volcano plot representing the differentially expressed lncRNA, where red represents up-regulation in HCC and green represents down-regulation (N = 424). **(B)** Heat map showing the DElncRNAs (only showing the 50 top lncRNAs). Red code: up-regulation in HCC; blue code: down-regulation. N: normal (N = 50); T: Tumor (N = 338). **(C)** The heat map of the expression of 7 lncRNAs in the high/low risk score group (N = 238). (**D)** Survival analysis of the high/low-risk group. (**E)** The time-dependent ROC curve of 1-year, 3-year, and 5-year for risk score model (N = 238). (For interpretation of the references to colour in this figure legend, the reader is referred to the Web version of this article.)

To establish a prognosis-related lncRNA signature, the present study investigated the differentially expressed lncRNAs first. The overview of this study was shown in Fig. 1. 3401 differentially expressed lncRNAs were screened from 14143 lncRNAs, and 6779 differentially expressed mRNAs were identified from 19695 mRNAs. The analysis of differentially expressed lncRNAs showed that the differential expression of lncRNAs in HCC tissues was mainly high expression (3275 high expression, low expression up to 126), implying an imbalance, while the heat map (only showing the first 50 lncRNAs) showed the similar trend. (Fig. 2A and B).

To further clarify the relationship between 3401 differentially expressed lncRNAs and the prognosis of patients, the cancer tissue samples of 338 cases of hepatocellular carcinoma were randomly divided into training group (n = 238) and verification group (n = 100) according to the proportion of 7:3. Then univariate Cox regression analysis was performed in the training group. It is notable that a total of 34 lncRNA were identified. Furthermore, a multi-factor Cox regression model was constructed in the training group, and the stepwise regression-backward method was used to repeatedly fit the model, from which the model with the lowest AIC value and the highest LR value was selected, and finally the 7-lncRNA risk score model (AL161937, LINC01063, AC145207, POLH-AS1, LNCSRLR, MKLN1-AS and AC105345) was obtained. These 7-lncRNAs were applied to constitute the risk model as follows: Risk Score = −4.13484 × AC105345 + 0.439897 × AL161937 + 0.569063 × LINC01063 + 0.598674 × AC145207 + 0.922016 × POLH-AS1 + 0.630741 × LNCSRLR +1.369449 × MKLN1-AS. The statistical information of the seven lncRNAs of the model was shown in Table 1, and the *p* values are all less than 0.05. It was indicated that lncRNA AC105345 played a protective role, while the other six lncRNAs served as risk factors, which was in accordance with the heat map (Fig. 2C). Interestingly, it was consistent with the results of unbalanced expression in previous differential lncRNA analysis (Fig. 2A and B). In order to initially evaluate the prognostic ability of the 7-lncRNA risk model in the training group, patients were divided into high-risk and low-risk groups according to the median risk score, followed by survival curve analysis. As expected, worse prognosis of the high-risk cohort was observed (Fig. 2D). Moreover, the timeROC curve of the model was constructed and the AUC value was evaluated. It was suggested that the model presented good prediction ability for 1-year (0.798), 3-year (0.797) and 5-year (0.798) OS (Fig. 2E).

**Table 1:** The features of 7 lncRNAs included in the risk model.

Gene name	Coefficient	Type	Down/up regulated	HR	95% CI		*p* value
					Lower	Upper	
AC105345.1	−4.13484	Protective	Down	0.016005	0.001234	0.20767	0.033902
AL161937.2	0.439897	Risky	Up	1.552547	0.915732	2.632211	0.03786
LINC01063	0.569063	Risky	Up	1.766611	1.066608	2.926019	0.018639
AC145207.5	0.598674	Risky	Up	1.819704	1.185717	2.792676	0.014482
POLH-AS1	0.922016	Risky	Up	2.514355	1.5384	4.109452	0.006804
LNCSRLR	0.630741	Risky	Up	1.879003	0.948559	3.722124	0.000731
MKLN1-AS	1.369449	Risky	Up	3.933187	2.037594	7.592266	0.000137

### 3.2 Verification of 7-lncRNAs risk model

**Fig. 3:**
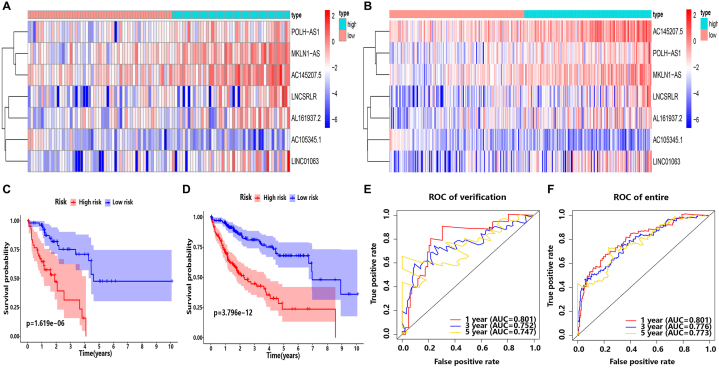
LncRNAs model verification and screening of independent clinical risk factors for overall survival. The heat map of the expression of 7 lncRNAs in the high/low risk score group in the verification group (N = 100) **(A)** and in the complete data set (N = 338) **(B)**. Survival analysis of the high/low-risk group in the verification group (N = 100) **(C)** and in the complete data set (N = 338) **(D)**. The time-dependent ROC curve of 1-year, 3-year, and 5-year for risk model in the verification group (N = 100) **(E)** and in the complete data set (N = 338) **(F)**.

To verify the prognostic ability of the risk model, heat map analysis, survival curve analysis and timeROC curve analysis were performed in the verification group (N = 100). The difference of lncRNA expression and survival probability in the high/low risk group were highly similar to those in the training group. The AUC values of 1-year, 3-year and 5-year ROC curve were 0.801, 0.752 and 0.747, respectively, indicating that the model deserved good prediction ability in the verification group (Fig. 3A, C, Fig.3E). Also, the similar results were obtained in the complete data set (Fig. 3B, D, Fig.3F). It is indicated that the risk model obtains high specificity and sensitivity in predicting prognosis.

**Fig. 4:**
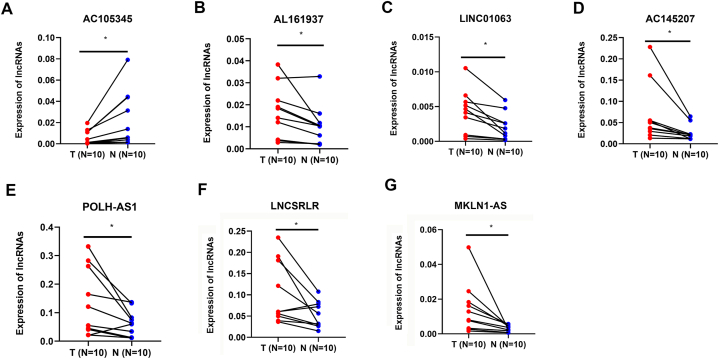
Validation of 7 lncRNAs expression in the risk model in 10 clinical samples: (**A**) AC105345.1 (**B**) AL161937.2 (**C**) LINC01063 (**D**) AC145207.5 (**E**) POLH-AS1 (**F**) LNCSRLR (**G**) MKLN1-AS.

To further verify the model in clinical samples, qPCR was conducted in 10 HCC specimens from Sun Yat-sen Memorial Hospital, Sun Yat-sen University. Expectedly, the differential expression of the 7 lncRNAs were in accordance with the database (Fig. 4).

**Fig. 5:**
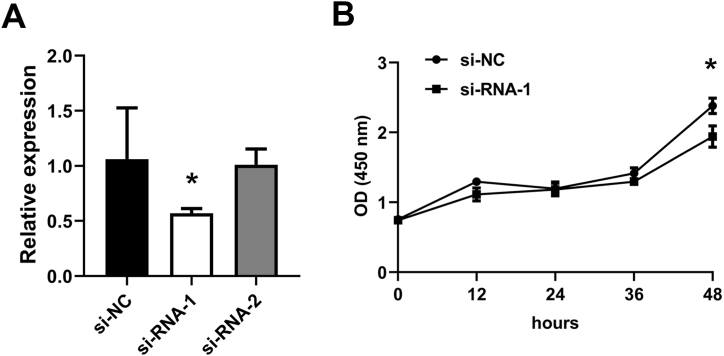
Inhibition of MKLN1-AS suppressed the proliferation potential of HepG2 cells. (**A**) Effective inhibition of MKLN1-AS expression. (**B**) Attenuation of MKLN1-AS expression prohibited cell growth of HepG2 cells.

To explore the biological role of 7 lncRNAs, MKLN1-AS was chosen for further study due to its markedly differential expression. Effective inhibition was observed by using siRNAs (Fig. 5A). Moreover, CCK8 assay was carried out to investigate the proliferation potential of MKLN1-AS. Interestingly, suppression of MKLN1-AS expression attenuated the proliferation ability (Fig. 5B). Taken together, MKLN1-AS may serve a oncogenic role as expected.

### 3.3 The risk score of 7-lncRNA model exerts as independent risk factors

**Fig. 6:**
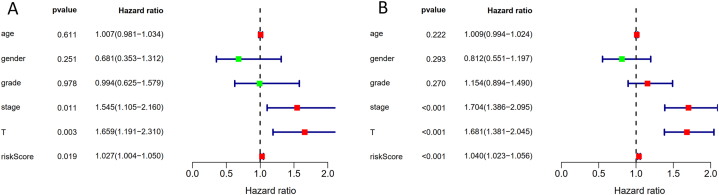
Analysis of independent clinical risk factors to the prognosis. It was indicated that stage, T stage and the risk score served an independent risk factor in the training group (N = 238) **(A)** and the complete data set (N = 338) **(B).**

To further evaluate the clinical significance of 7-lncRNA model and identify the possible independent clinical risk factors, Cox regression analysis was carried out in training group and complete data set by combining clinical data. Interestingly, the risk score, stage (AJCC TNM) and T stage served as risk factors both in training group (Fig. 6A) and complete data set (Fig. 6B). Considering that AJCC TNM consists of T stage, therefore, it was suggested that both Risk Score and AJCC TNM stages were independent clinical risk factors in hepatocellular carcinoma.

**Fig. 7:**
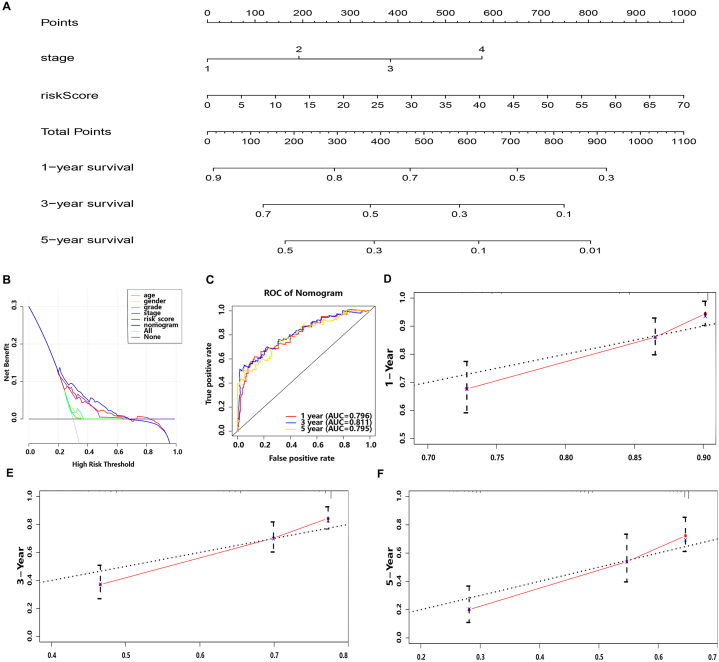
Assessment of the clinical prognostic value of the nomogram. (**A)** Development of the clinical prognostic nomogram to predict 1-, 3-, and 5-year survival. The vertical scale presented the independent clinical risk factor that has been screened out. The horizontal scale exerted the corresponding score. **(B)** DCA curve incorporating all clinical factors, risk model and nomogram. **(C)** 1-year, 3-year, 5-year time-dependent ROC curve for nomogram. The correction curve of predicted survival rate of nomogram for 1-year **(D)**, 3-year **(E)** and 5-year **(F)**.

To make accurately prognostic prediction and facilitate personalized treatment for each patient, a nomogram consisting of screened independent clinical risk factors (including 7-lncRNA risk score model) was constructed (Fig. 7A). DCA, timeROC curve, C-index and calibration curve were performed to evaluate the prediction ability and the accuracy of the nomogram. Interestingly, the DCA curve combining all clinical factors, risk model and nomogram implied that the net return of nomogram was better than risk score or TNM stage itself, and also better than other individual clinical factors (Fig. 7B). It was suggested that the nomogram served a better predictive effect. The AUC values of 1-year, 3-year and 5-year ROC curve are 0.796, 0.811 and 0.795 respectively (Fig. 7C), and the C-index is 0.696 (95%CI:0.644–0.767, *p* < 0.001). In the calibration curve, the predicted value of the nomogram in 1 year, 3 years and 5 years has a high coincidence with the actual value, revealing that the line chart obtained a good accuracy (Fig. 7D and F). These results indicated that the nomogram presented a good ability for prognostic prediction, which was consistent with the result of DCA curve.

**Fig. 8:**
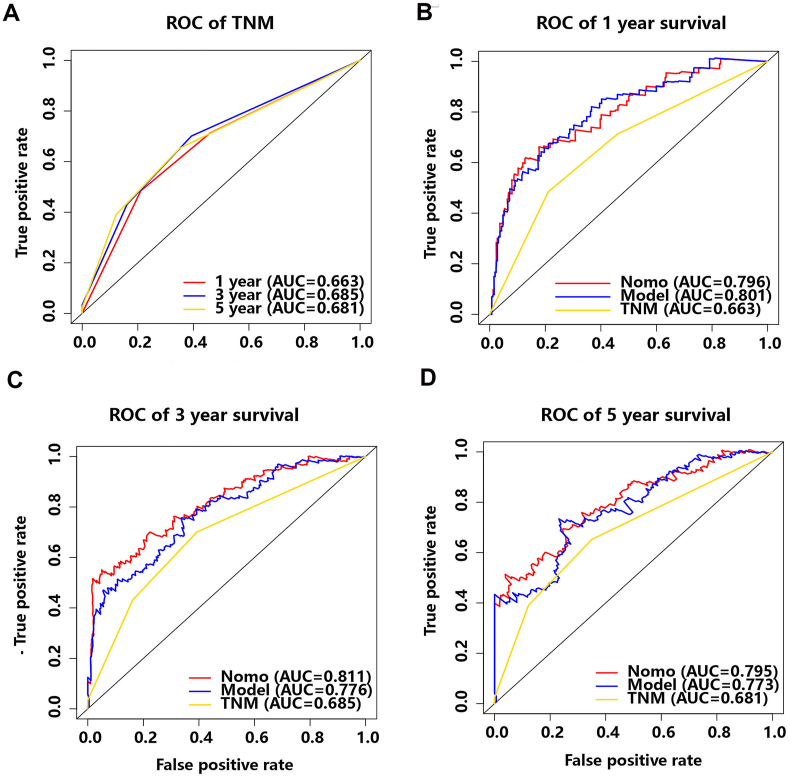
Comparison of time-dependent ROC curve for AJCC TNM stage. **(A)** 1-year, 3-year, 5-year time-dependent ROC curve for AJCC TNM stage. **(B**–**D)** Comparison of the 1-year **(B)**, 3-year **(C)**, and 5-year time-dependent ROC curve **(D)** among nomogram, 7- lncRNA risk model and AJCC TNM stage.

As proven above, the risk model and the nomogram present a good potential for prognosis prediction. To further evaluate the clinical value of the both, the study first investigated prognostic potential of AJCC TNM stage, which served as an ideal model. The time-dependent ROC curve of the AJCC TNM stage in TCGA-LIHC cohort for 1-, 3- and 5-year were constructed (Fig. 8A). Meanwhile, ROC curve among nomogram, risk model and AJCC TNM stage for 1- year (Fig. 8B), 3-year (Fig. 8C) and 5-year (Fig. 8D) was established. Interestingly, AUC on the risk model and the nomogram showed that both of them had a better accuracy in OS than AJCC TNM stage in 1-year survival (0.801, 0.796), 3-year survival (0.776, 0.811) and 5-year survival (0.773, 0.795) (Table 2).

**Table 2:** Comparison of the AUC value in three different prediction methods.

Methods	Mean	SD	SE	95%CI		t	*p* value
				lower	upper		
Nomogram vs TNM	0.124	0.01	0.006	0.101	0.148	22.411	0.002
Model vs TNM	0.107	0.027	0.016	0.04	0.174	6.902	0.02
Nomogram vs Model	0.017	0.02	0.012	−0.033	0.068	1.471	0.279

### 3.4 Bioinformatic functional annotation of the risk model

**Fig. 9:**
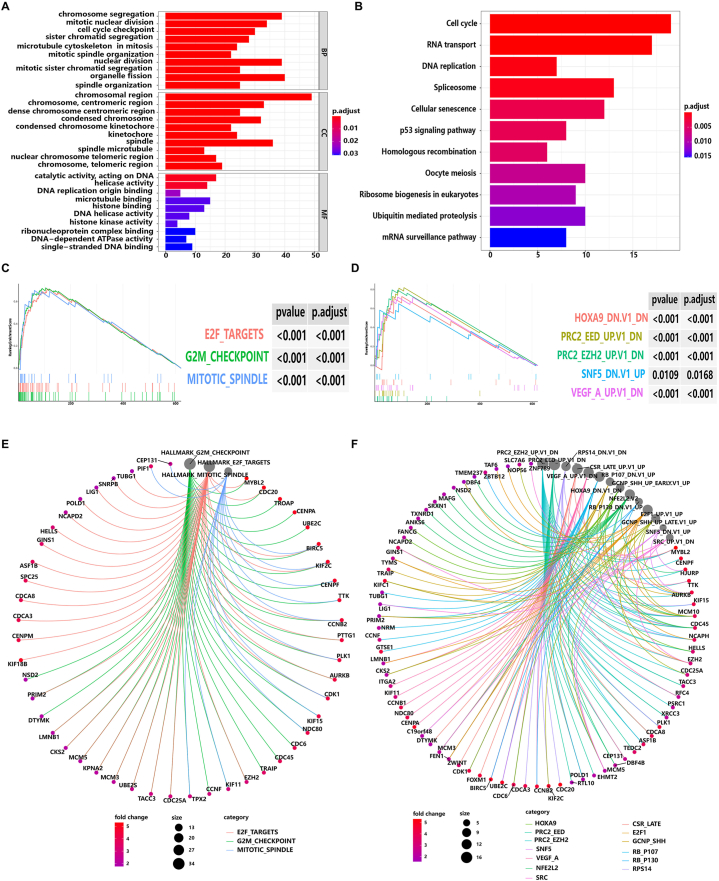
Bioinformatic analysis of the risk model. **(A)** GO enrichment analysis of signal pathways. BP: biological process. CC: cell component. MF: molecular function. **(B)** KEGG enrichment analysis. **(C)** GSEA enrichment map of classic pathway. **(D)** GSEA enrichment map of gene pathway. **(E)** Specific gene enrichment diagram of classic pathway. **(F)** Specific gene enrichment diagram of cancer gene pathway.

In order to explore the potential biological roles of the seven lncRNA of the risk model on the prognosis of HCC, KEGG and GO analysis were performed. Impressively, DEGs between the high-risk and the low-risk alignment were related to cell cycle, p53 signaling pathway, RNA transport and DNA replication (Fig. 9A and B). Moreover, GSEA was performed and found that the classical pathway (hallmarks) was rich in E2F targets, G2M checkpoints and mitotic spindles, which were related to the cell cycle regulatory network (Fig. 9C and E), which was consistent with the results of KEGG and GO analysis. Also, it was implied that cancer-associated gene pathways were enriched in HOXA9, PRC2-EED, PRC2-EZH2, SNF5, VEGF-A (Fig. 9D and F), which exerted as a vital role in transcriptional regulation, epigenetic regulation and tumor angiogenesis. These biomarkers provide a new potential direction for the molecular mechanism research in HCC.

### 3.5 The risk model was associated with immune cell infiltration

**Fig. 10:**
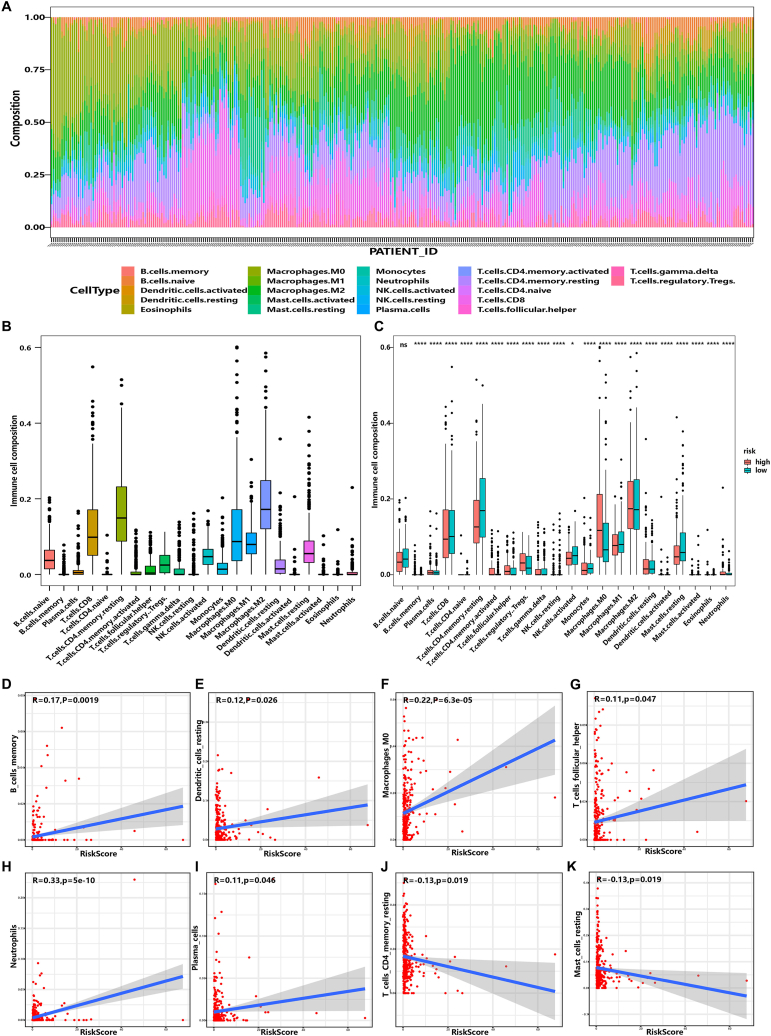
The distribution of immune microenvironment in HCC between the high-risk and low-risk groups. **(A)** Immune cell infiltration pattern in the TCGA-LIHC cohort. **(B)** Differential distribution of the 22 kinds of immune cells in TCGA-LIHC cohort. **(C)** Remarkably differential distribution of the immune cells was identified. 20 out of the 22 types of immune cells was confirmed in the high-risk group. **(D**–**K)** Relationship between risk score and the immune infiltrating cells. A positive correlation between the risk score and the expression of memory B cells **(D)**, resting Dendritic cells **(E)**, Macrophages-M0 **(F),** follicular helper T cell **(G)**, Neutrophils **(H)** and Plasma cells **(I)**, while a negative correlation between the risk score and resting CD4^+^ memory T cell **(J)** and resting Mast cells **(K)** was detected.

As presented above, the high-risk group of the risk model suffered a worse prognosis. Meanwhile, the ability of immune evasion had been identified as a hallmark of cancer. To explore whether the risk model made a contribution to the immune cell infiltration, CIBERSORT algorithm was employed to assess the proportion of the 22 kinds of immune cell infiltration in tumor sample in TCGA-LIHC cohort. Intriguingly, it was suggested that the overall distribution proportion of the 22 types of immune cells in different samples was heterogeneous (Fig. 10A) and the higher infiltration of CD8^+^ T cell, resting CD4^+^ memory T cell, Macrophages-M0, Macrophages-M1 in 338 TCGA-LIHC cohort samples was observed (Fig. 10B). 22 kinds of immune cell infiltration ratios were further analyzed in combination with the model, and the patients were divided into high risk group and low risk group by the median of risk score. Markedly, 20 out of 22 types of the immune cells differential distribution was identified between high- and low-risk alignment (*p* < 0.001, Fig. 10C). Among the 20 immune cells, a lower proportions of resting memory CD4^+^ T cell, CD8^+^ T cell, mast resting cells, and a higher proportions of regulatory T cells, M0 macrophages, follicular helper T cells in the high-risk group were identified. In order to explore whether there was a correlation between these related immune cells and risk score, the expression of Risk Score and immune cells was analyzed by Pearson. Intriguingly, the results revealed that a positive correlation between the risk score and the expression of memory B cells (Fig. 10D), resting Dendritic cells (Fig. 10E), Macrophages-M0 (Fig. 10F), follicular helper T cell (Fig. 10G), Neutrophils (Fig. 10H) and Plasma cells (Fig. 10I) was observed. Meanwhile, a negative correlation between the risk score and resting CD4^+^ memory T cell (Fig. 10J) and resting Mast cells (Fig. 10K) was detected. Taken together, the risk model was associated with immune cell infiltration. The seven lncRNAs of the model may make a contribution to regulate the proportion of immune infiltrating cells around tumor cells, thus affecting the occurrence, development and prognosis of hepatocellular carcinoma, which also provides a novel direction to further study the molecular mechanism of HCC carcinogenesis.

## 4 Discussion

For better evaluate the prognosis of HCC patients, the present study used R software to analyze high-throughput lncRNA data of HCC patients to obtain a 7-lncRNA risk model in this study, and its fine sensitivity and specificity were proved after evaluation. The risk model and visual nomogram constructed and validated performed outstanding predictive accuracy, calibration and applicability. Ultimately, it initially explored the biological signaling pathways and immune infiltration-related mechanisms implicated in the model's ability to affect the prognosis.

For the 7 lncRNAs included in the model of this study, AC105345.1 is low-expressed in cancer tissues, as a protective factor for hepatocellular carcinoma, while the remaining 6 lncRNAs are all high-risk factors, were correlated with worse prognosis in HCC patients. Among these lncRNAs, an extremely finite number have been probed by previous molecular experiments about their biological function in the pathology of HCC. Consistent with the results of this study, another study [33] also implied that MKLN1-AS was up-regulated in HCC tissues and the cell lines, which could increase hepatoma derived growth factor (HDGF), thereby restraining tumor cell apoptosis and boosting the proliferation, migration and invasion of HCC, and then being distinctly interrelated to the shortening of the overall survival of patients. Recently, another study revealed that the role of LINC01063 in HCC might be related to autophagy [34], and specifically had relationship with the tumor suppressor p53 binding protein 1 (TP53BP1), whose recruitment could trigger autophagy by tumor self-DNA damage response [35], and charged multivesicular protein 4B (CHMP4B), whose recruitment was critical in the later stages of mitochondrial phagosome formation [36]. Besides, the effect of AC145207.5 on the prognosis of HCC has also been discovered to be relevant to the autophagy mechanism recently [37], and it was further unfolded to affect the cell cycle by regulating glycolysis [38] and step in the level of infiltration of tumor-infiltrating immune cells [39], due to which it accelerated cancer cell proliferation and disease progression. The remaining 4 lncRNAs in the 7-lncRNA risk model related to HCC have not yet seen reports, which means that further exploration is necessary, and the similarities in the previous experimental results of model related lncRNAs affecting the prognostic mechanism of HCC in many articles also offers clearer objects and directions for basic experimental verification, such as in autophagy, glycolysis, or immune infiltration, etc.

For the purpose of investigating the possible mechanisms of 7 lncRNAs affecting the prognosis of HCC, the present study performed KEGG, GO and GSEA analysis, and CIBERSORT immune infiltration analysis. Among them, the pathways enriched by KEGG and GO mainly involve cell cycle, spliceosome, RNA transport, and P53 pathway. With reference to the existing or related reports on these pathways and lncRNA, the clues to the relevant mechanisms we are concerned about may be got. For example, QIN G et al. found that the enhancement of the interaction between p53 and heterogeneous ribonucleoprotein K could be achieved by the combination of lncRNA-PSTAR and heterogeneous ribonucleoprotein K, which further leaded to the accumulation and transactivation of p53 and induced cell cycle arrest to restrain the proliferation and metastasis of HCC cells [40]. In addition, splicing factor 3B subunit 1 was detected as a vital splicing factor overexpressing in a variety of tumors, after being blocked, it could attenuate tumor aggressiveness and the expression of oncogenic splice variants, and raise overall survival [41]. Different from KEGG and GO enrichment analysis, GSEA brings in all co-expressed genes rather than differential genes and is able to detect pathways that have little change but are important for prognosis. In the classic pathway, similar to the results of the KEGG and GO enrichment analysis, the cell cycle control members such as E2F targets, G2M checkpoint, and mitotic spindle are enriched, while the cancer gene pathway is enriched in HOXA9, PRC2-EED, SNF5, VEGF-A, and PRC2-EZH2 etc. There is already a lot of evidence to verify the role of these pathways or genes, as show below. As an important transcription factor, E2F1, E2F3, and E2F8 have been detected to be significantly up-regulated in human HCC. Among them, E2F1 inhibited c-Myc-induced apoptosis through PIK3CA/Akt/mTOR pathway while E2F8 increased the proportion of S-phase cells by up-regulating the expression of cyclinD1, next promoted the occurrence and development of HCC [[42], [43], [44], [45], [46]]. G2M checkpoint is one of the members of DNA damage checkpoint, preventing DNA damaged cells in G2 phase from entering the final checkpoint of mitosis (M phase) [47]. In HCC, more than 70% of activated cyclin-dependent kinases forms a complex with cyclinB, and then, similar effect to the E2F family, they are involved in the block regulation of the G2M checkpoint [48,49]. Different from the most common abnormal upregulation in acute myeloid leukemia [50], the HOXA9 gene is highly methylated and its downstream mRNA expression is significantly decreased in HCC, presenting the role of tumor suppressor genes [51]. In addition, studies have shown that SNF5 was also an important epigenetic factor that acted on the regulatory process of tumors [52,53], and its knockout could bring about tumor progression [54]. According to the properties of VEGF-A in promoting tumor angiogenesis and increasing vascular permeability [55,56]**,** there is no surprise that VEGF-A is significantly overexpressed in cell liver cancer, and the expression level in advanced liver cancer is higher than that in early stage [[56], [57], [58]]. All in all, the above studies hint that the 7 lncRNAs inside risk model may directly or indirectly participate in cell cycle pathway regulation, transcription regulation, epigenetic regulation, tumor angiogenesis and other biological processes through one or more mechanisms, and were remarkably associated with occurrence, proliferation and metastasis of HCC, which ultimately affect the prognosis of HCC. This also provides a new direction for the specific mechanism research in the future.

Studies have shown that tumor microenvironment can affect the level of gene expression in tumor tissue, thus affecting the occurrence, development and metastasis of tumor, and the phenomenon of immune escape in tumor microenvironment is closely related to the individual efficacy of tumor immunotherapy [59,60]. The significantly different expression levels of 20 kinds of immune cells inextricably linked to tumor microenvironment between the high-risk patients and the low-risk patients were displayed. Lower proportions of resting memory CD4^+^ T cell，CD8+ T cell and mast resting cells, with higher proportions of regulatory T cells, M0 macrophages and follicular helper T cells was investigated in the high-risk group, implying that the immune cell subtypes infiltration might unfold a crucial impact on the prognosis of HCC patients. Similar results were proven [61] that the immune tolerance of the high-risk patients might bridge with iron death to work reasonably well in tumorigenesis and immune escape of HCC patients. Currently, emerging studies have confirmed the important role of tumor infiltrating lymphocytes including tumor associated macrophages, regulatory T cells in triggering immune evasion in the process of HCC development [62]. Meanwhile, it was showed that macrophages could enhance the invasion ability of liver cancer by destabilizing adhesion junctions after activation [63]. Besides, it was shown that the local immune response of HCC patients might be contributed by tumor-infiltrating CD4^+^CD25^+^ regulatory T lymphocytes which suppressed the activity and proliferation of antitumor effector CD4^+^ and CD8^+^ T cells, thus, making an effort on the metastasis and recurrence rate of liver cancer [64]. Moreover, excluding quantitative changes, the CD8^+^ lymphocytes infiltrating HCC performed impaired function that lacked cytotoxic markers, where we called this functionally impaired state as “T cell depletion”, failing to effectively eliminate cancer cells [65,66]. Coincidentally, it was demonstrated that the better prognosis in the early stage may be due to the better anti-tumor effect caused by the cellular immunity of CD4^+^ and CD8^+^ T lymphocytes. Interestingly, CD8^+^ T lymphocyte worked predominantly comparing to CD4^+^ T lymphocyte, and the humoral immunity of B cells that facilitated early lymphoid follicles formation [67]. Accordingly, it is boldly speculated that 7 lncRNAs in the risk model may become inducers or participate in intermediate processes in some way to affect the abundance of immune infiltrating cells around tumor cells, especially in T lymphocytes, and then have an impact on the progression and survival of HCC patients.

## 5 Conclusion

The present study constructed and validated a novel 7-lncRNAs signature (AC105345, LINC01063, AC145207, AL161937, POLH-AS1, LNCSRLR and MKLN1-AS) for HCC prognosis prediction. This signature obtained a good applicability and accuracy in predicting the prognosis of HCC. Furthermore, the nomogram was constructed by 7-lncRNAs signature and selected independent clinical risk factors, and the analysis showed that the accuracy of 7-lncRNAs signature and nomogram in OS staging was better than that in classical AJCC TNM staging. In addition, this study preliminarily explored the potential biological effects of 7 lncRNA on the prognosis of hepatocellular carcinoma. The study showed that they play an important role in cell cycle regulation, p53 signal pathway, transcriptional regulation, epigenetic regulation, tumor angiogenesis and so on. Finally, the results revealed that most of the immune infiltrating cells were highly correlated with 7-lncRNAs signature, and positively correlated with the expression of memory B cells, resting Dendritic cells, Macrophages-M0, follicular helper T cell, Neutrophils and Plasma cells, and negatively correlated with resting CD4^+^ memory T cell and resting Mast cells. To sum up, the 7-lncRNAs markers constructed in this study have a vital role in predicting the prognosis, and have made a preliminary exploration on the biological role of the markers, which provides new ideas for the study of the occurrence and development of HCC.

### 5.1 Research limitations

1. The lncRNA selected in this study was obtained from the public database TCGA, and the number of samples included was only 338 cases. The sample size and demography had limitations, and the conclusions were obtained from statistical calculation and inference, and also had some limitations.
2. In this study, the lncRNA of 10 cases of HCC cancer and paracancerous tissues were analyzed by RT-PCR, but the sample size was limited and there may be statistical errors.

## Author contribution statement

Fenting Huang; Shineng Zhang: Conceived and designed the experiments.

Junchi Qu: Performed the experiments; Analyzed and interpreted the data; Wrote the paper.

Di Tao: Analyzed and interpreted the data; Contributed reagents, materials, analysis tools or data; Wrote the paper.

Wei Huang; Liting Lu: Analyzed and interpreted the data; Contributed reagents, materials, analysis tools or data.

Junming Fan: Contributed reagents, materials, analysis tools or data.

## Data availability statement

The original contributions presented in the study are included in the article/Supplementary Materials, further inquiries can be directed to the corresponding author.

## Declaration of competing interest

The authors declare that they have no known competing financial interests or personal relationships that could have appeared to influence the work reported in this paper.

## References

[1] Ferlay J. (2015). Cancer incidence and mortality worldwide: sources, methods and major patterns in GLOBOCAN 2012. Int. J. Cancer.

[2] Gravitz L. (2014). Liver cancer. Nature.

[3] Sung H. (2021). Global cancer statistics 2020: GLOBOCAN estimates of incidence and mortality worldwide for 36 cancers in 185 countries. Ca-Canc. J. Clin..

[4] Yao F.Y., Fidelman N. (2016). Reassessing the boundaries of liver transplantation for hepatocellular carcinoma: where do we stand with tumor down-staging?. Hepatology.

[5] Chen W. (2016). Cancer statistics in China, 2015. CA A Cancer J. Clin..

[6] Zhang B.H., Yang B.H., Tang Z.Y. (2004). Randomized controlled trial of screening for hepatocellular carcinoma. J. Cancer Res. Clin. Oncol..

[7] Tellapuri S. (2018). Staging systems of hepatocellular carcinoma: a review. Indian J. Gastroenterol..

[8] Li H. (2016). Comparison of the predictive values of eight staging systems for primary liver cancer in prognosis of combined hepatocellular-cholangiocellular carcinoma patients after surgery. Zhongguo Yi Xue Ke Xue Yuan Xue Bao.

[9] Huarte M., Rinn J.L. (2010). Large non-coding RNAs: missing links in cancer?. Hum. Mol. Genet..

[10] Rinn J.L., Chang H.Y. (2012). Genome regulation by long noncoding RNAs. Annu. Rev. Biochem..

[11] Zhao X. (2018). Long noncoding RNA n339260 promotes vasculogenic mimicry and cancer stem cell development in hepatocellular carcinoma. Cancer Sci..

[12] Liu Z. (2020). Construction of lncRNA-associated ceRNA networks to identify prognostic lncRNA biomarkers for glioblastoma. J. Cell. Biochem..

[13] Ulitsky I. (2011). Conserved function of lincRNAs in vertebrate embryonic development despite rapid sequence evolution. Cell.

[14] Hezroni H. (2015). Principles of long noncoding RNA evolution derived from direct comparison of transcriptomes in 17 species. Cell Rep..

[15] Quinn J.J. (2016). Rapid evolutionary turnover underlies conserved lncRNA-genome interactions. Genes Dev..

[16] Prensner J.R., Chinnaiyan A.M. (2011). The emergence of lncRNAs in cancer biology. Cancer Discov..

[17] Prensner J.R. (2011). Transcriptome sequencing across a prostate cancer cohort identifies PCAT-1, an unannotated lincRNA implicated in disease progression. Nat. Biotechnol..

[18] Ritchie M.E. (2015). Limma powers differential expression analyses for RNA-sequencing and microarray studies. Nucleic Acids Res..

[19] Cox B.D.R. (1972). Regression models and life-tables. J. Royal Stat. Soci..

[20] Akaike H. (1974). A new look at the statistical model identification. IEEE Trans. Automat. Control.

[21] Akaike H. (1998).

[22] Lorent M., Giral M., Foucher Y. (2014). Net time-dependent ROC curves: a solution for evaluating the accuracy of a marker to predict disease-related mortality. Stat. Med..

[23] Wang S. (2020). An eight-CircRNA assessment model for predicting biochemical recurrence in prostate cancer. Front. Cell Dev. Biol..

[24] Blanche P., Dartigues J.F., Jacqmin-Gadda H. (2013). Estimating and comparing time-dependent areas under receiver operating characteristic curves for censored event times with competing risks. Stat. Med..

[25] Iasonos A. (2008). How to build and interpret a nomogram for cancer prognosis. J. Clin. Oncol..

[26] Fitzgerald M., Saville B.R., Lewis R.J. (2015). Decision curve analysis. JAMA.

[27] Kanehisa M., Goto S. (2000). KEGG: kyoto encyclopedia of genes and genomes. Nucleic Acids Res..

[28] Ashburner M. (2000). Gene Ontology: tool for the unification of biology. Nat. Genet..

[29] Yu G. (2012). clusterProfiler: an R package for comparing biological themes among gene clusters. OMICS.

[30] Subramanian A. (2005). Gene set enrichment analysis: a knowledge-based approach for interpreting genome-wide expression profiles. Proc. Natl. Acad. Sci. U. S. A.

[31] Mootha V.K. (2003). PGC-1alpha-responsive genes involved in oxidative phosphorylation are coordinately downregulated in human diabetes. Nat. Genet..

[32] Newman A.M. (2019). Determining cell type abundance and expression from bulk tissues with digital cytometry. Nat. Biotechnol..

[33] Gao W. (2020). Long non-coding RNA MKLN1-AS aggravates hepatocellular carcinoma progression by functioning as a molecular sponge for miR-654-3p, thereby promoting hepatoma-derived growth factor expression. Int. J. Mol. Med..

[34] Deng X. (2020). Identification of a five-autophagy-related-lncRNA signature as a novel prognostic biomarker for hepatocellular carcinoma. Front. Mol. Biosci..

[35] Sharma A. (2018). USP14 regulates DNA damage repair by targeting RNF168-dependent ubiquitination. Autophagy.

[36] Zhen Y. (2020). ESCRT-mediated phagophore sealing during mitophagy. Autophagy.

[37] Wang M. (2021). Construction of prognostic model of autophagy-related LncRNA in hepatocellular carcinoma. J. Xi'an Jiaot. Univer. Med. Sci.

[38] Xia X. (2021). Identification of glycolysis-related lncRNAs and the novel lncRNA WAC-AS1 promotes glycolysis and tumor progression in hepatocellular carcinoma. Front. Oncol..

[39] Zhou P. (2021). Construction of an immune-related six-lncRNA signature to predict the outcomes, immune cell infiltration, and immunotherapy response in patients with hepatocellular carcinoma. Front. Oncol..

[40] Qin G. (2020). Long noncoding RNA p53-stabilizing and activating RNA promotes p53 signaling by inhibiting heterogeneous nuclear ribonucleoprotein K deSUMOylation and suppresses hepatocellular carcinoma. Hepatology.

[41] Seiler M. (2018). Somatic mutational landscape of splicing factor genes and their functional consequences across 33 cancer types. Cell Rep..

[42] Deng Q. (2010). E2F8 contributes to human hepatocellular carcinoma via regulating cell proliferation. Cancer Res..

[43] Liu L. (2013). Genetic variants in pseudogene E2F3P1 confer risk for HBV-related hepatocellular carcinoma in a Chinese population. J. Biomed. Res..

[44] Ladu S. (2008). E2F1 inhibits c-Myc-driven apoptosis via PIK3CA/Akt/mTOR and COX-2 in a mouse model of human liver cancer. Gastroenterology.

[45] Huang Y. (2013). HBV core promoter mutations promote cellular proliferation through E2F1-mediated upregulation of S-phase kinase-associated protein 2 transcription. J. Hepatol..

[46] Trimarchi J.M., Lees J.A. (2002). Sibling rivalry in the E2F family. Nat. Rev. Mol. Cell Biol..

[47] Xu B. (2002). Two molecularly distinct G(2)/M checkpoints are induced by ionizing irradiation. Mol. Cell Biol..

[48] Dimova D.K., Dyson N.J. (2005). The E2F transcriptional network: old acquaintances with new faces. Oncogene.

[49] Li K.K. (2002). Activation of cyclin-dependent kinases CDC2 and CDK2 in hepatocellular carcinoma. Liver.

[50] Collins C.T., Hess J.L. (2016). Role of HOXA9 in leukemia: dysregulation, cofactors and essential targets. Oncogene.

[51] Kuo C.C. (2014). Frequent methylation of HOXA9 gene in tumor tissues and plasma samples from human hepatocellular carcinomas. Clin. Chem. Lab. Med..

[52] Wu J.N., Roberts C.W. (2013). ARID1A mutations in cancer: another epigenetic tumor suppressor?. Cancer Discov..

[53] Shain A.H., Pollack J.R. (2013). The spectrum of SWI/SNF mutations, ubiquitous in human cancers. PLoS One.

[54] Wilson B.G. (2010). Epigenetic antagonism between polycomb and SWI/SNF complexes during oncogenic transformation. Cancer Cell.

[55] Dhar D.K. (2002). Requisite role of VEGF receptors in angiogenesis of hepatocellular carcinoma: a comparison with angiopoietin/Tie pathway. Anticancer Res..

[56] Park Y.N. (2000). Increased expression of vascular endothelial growth factor and angiogenesis in the early stage of multistep hepatocarcinogenesis. Arch. Pathol. Lab Med..

[57] Fernández M. (2009). Angiogenesis in liver disease. J. Hepatol..

[58] Tseng P.L. (2008). Overexpression of VEGF is associated with positive p53 immunostaining in hepatocellular carcinoma (HCC) and adverse outcome of HCC patients. J. Surg. Oncol..

[59] D'Arcy M.E. (2019). Survival after a cancer diagnosis among solid organ transplant recipients in the United States. Cancer.

[60] O'Donnell J.S., Teng M.W.L., Smyth M.J. (2019). Cancer immunoediting and resistance to T cell-based immunotherapy. Nat. Rev. Clin. Oncol..

[61] Xu Z. (2021). Construction of a ferroptosis-related nine-lncRNA signature for predicting prognosis and immune response in hepatocellular carcinoma. Front. Immunol..

[62] Lurje I. (2021). In situ vaccination as a strategy to modulate the immune microenvironment of hepatocellular carcinoma. Front. Immunol..

[63] Coudert J.D. (2005). Altered NKG2D function in NK cells induced by chronic exposure to NKG2D ligand-expressing tumor cells. Blood.

[64] Unitt E. (2005). Compromised lymphocytes infiltrate hepatocellular carcinoma: the role of T-regulatory cells. Hepatology.

[65] Wherry E.J. (2011). T cell exhaustion. Nat. Immunol..

[66] Thommen D.S., Schumacher T.N. (2018). T cell dysfunction in cancer. Cancer Cell.

[67] Wada Y. (1998). Clinicopathological study on hepatocellular carcinoma with lymphocytic infiltration. Hepatology.

