# Supplementary material for: Assessment of prognostic role of a novel 7-lncRNA signature in HCC patients
Source: Heliyon. 2023 Jul 20;9(8):e18493. doi: 10.1016/j.heliyon.2023.e18493 (PMC10382640; doi:10.1016/j.heliyon.2023.e18493)
Supplement: Multimedia component 1 [file mmc1.docx]

**Supplementary Table 1** Clinical characteristics of HCC patients.

| Character | Training dataset | Verification dataset | Entire dataset | P value |
| --- | --- | --- | --- | --- |
|  | n=238 | n=100 | n=338 |  |
| **Age (year)** | |  |  | 0.827118 |
| ≥65 | 98（41.18%） | 32（32.00%） | 130（38.46%） |  |
| ＜65 | 140（58.82%） | 68（68.00%） | 208（61.54%） |  |
| **Gender** |  |  |  | 0.777999 |
| female | 78（32.77%） | 29（29.00%） | 107（31.66%） |  |
| male | 160（67.23%） | 71（71.00%） | 231（68.34%） |  |
| **Stage** |  |  |  | 0.964181 |
| I | 120（50.42%） | 48（48.00%） | 168（49.70%） |  |
| II | 56（23.53%） | 27（27.00%） | 83（24.56%） |  |
| III | 59（24.79%） | 24（24.00%） | 83（24.56%） |  |
| IV | 3（1.26%） | 1（1.00%） | 4（1.18%） |  |
| **T** |  |  |  | 0.934173 |
| T1 | 122（51.26%） | 48（48.00%） | 170（50.30%） |  |
| T2 | 56（23.53%） | 28（28.00%） | 84（24.85%） |  |
| T3 | 53（22.27%） | 21（21.00%） | 74（21.89%） |  |
| T4 | 7（2.94%） | 3（3.00%） | 10（2.96%） |  |
| **M** |  |  |  | 0.684871 |
| M0 | 179（75.21%） | 80（80.00%） | 259（76.63%） |  |
| M1 | 2（0.84%） | 1（1.00%） | 3（0.89%） |  |
| MX | 57（23.95%） | 19（19.00%） | 76（22.49%） |  |
| **N** |  |  |  | 0.751862 |
| N0 | 171（71.85%） | 76（76.00%） | 247（73.08%） |  |
| N1 | 3（1.26%） | 1（1.00%） | 4（1.18%） |  |
| N2 | 0（0.00%） | 0（0.00%） | 0（0.00%） |  |
| N3 | 0（0.00%） | 0（0.00%） | 0（0.00%） |  |
| NX | 64（26.89%） | 23（23.00%） | 87（25.74%） |  |
