# Supplementary material for: Assessment of prognostic role of a novel 7-lncRNA signature in HCC patients
Source: Heliyon. 2023 Jul 20;9(8):e18493. doi: 10.1016/j.heliyon.2023.e18493 (PMC10382640; doi:10.1016/j.heliyon.2023.e18493)
Supplement: Multimedia component 2 [file mmc2.docx]

**Supplementary Table 2** The sequence of the primers.

| **Primer** | **Sequence (5' to 3')** |
| --- | --- |
| GAPDH-F | ACCCACTCCTCCACCTTTGAC |
| GAPDH-R | TGTTGCTGTAGCCAAATTCGTT |
| LNCSRLR-F | CACCTGGTATGATGTAGATGCC |
| LNCSRLR-R | TTGCACTGGTAGGAACGAAC |
| MKLN1-AS-F | GCCAATGTCCTATCTCAGGG |
| MKLN1-AS-R | TTACACCTCAGACCCAGTGT |
| POLH-AS1-F | GCAGCCTCTAGCTTGACATA |
| POLH-AS1-R | CCAGGGAAGCTTGTGACTTA |
| AC105345-F | CCCCTCTCTTTTGAGCAGATGA |
| AC105345-R | CGACGCATCTGTGTTCTCTT |
| LINC01063-F | GGCAGTTCAGCCACAATCAG |
| LINC01063-R | CCAATCACCTTCCAGGCTCA |
| AL161937-F | CTGGATGCTGTGCTGAAAAC |
| AL161937-R | GCATCTGGGCTCTCATCATC |
| AC145207-F | ATTGACTGGCCAAGCATTTG |
| AC145207-R | ACATGATCACAGACAAGCTGA |
